# The postbiotic of hawthorn-probiotic ameliorates constipation by multi-pathway inhibition of PANoptosis in intestinal epithelial cells

**DOI:** 10.3389/fimmu.2025.1622619

**Published:** 2025-09-19

**Authors:** Shiying Chen, Jieling Lin, Mengxia Huang, Shi Lin, Yu Wei, Cong Zhang, Yali Huang

**Affiliations:** ^1^ Research Centre of Basic Integrative Medicine, School of Basic Medical Sciences, Guangzhou University of Chinese Medicine, Guangzhou, Guangdong, China; ^2^ Gastrointestinal Surgery, The First Clinical Medical School, Guangzhou University of Chinese Medicine, Guangzhou, China; ^3^ Department of Gastroenterology, The First People's Hospital of Foshan (Foshan Hospital Affiliated to Southern University of Science and Technology), School of Medicine, Southern University of Science and Technology, Guangdong, China

**Keywords:** postbiotic, hawthorn, probiotic, constipation, apoptosis, pyroptosis, inflammation, immune modulation

## Abstract

**Background:**

Constipation is a common gastrointestinal disorder with significant public health implications, particularly among aging populations. Current treatment options often exhibit limited efficacy and may have potential side effects, underscoring the necessity for safer and more effective alternatives. Postbiotics, which are bioactive metabolites derived from probiotics, have emerged as promising therapeutic agents due to their stability, safety, and multifunctional properties. Postbiotic of hawthorn-probiotic has demonstrated potential in alleviating constipation; however, its mechanisms-particularly regarding the regulation of intestinal epithelial cell apoptosis and the gut microenvironment-remain to be fully elucidated.

**Methods:**

This study employed murine models of loperamide-induced constipation and *in vitro* assays using NCM460 intestinal epithelial cells. Conditioned medium mimicking the gut microenvironment was prepared from colonic contents of different groups of mice. Cell viability and apoptosis levels were assessed using flow cytometry and fluorescence microscopy, while molecular pathways were analyzed through RT-qPCR and Western blot techniques. Network pharmacology was employed to integrate transcriptomic data for identifying core targets and pathways.

**Results:**

Postbiotic of hawthorn-probiotic significantly downregulated apoptotic (CASP3, CASP9) and pyroptotic (NLRP3, GSDMD, CASP1) pathway components while restoring the balance of pro- and anti-apoptotic proteins (Bax/Bcl-2). Additionally, this postbiotic selectively modulated immune responses by reducing Th2/Th17-type cytokines (IL-2, IL-17A), without affecting Th1-type responses. Network analysis revealed that the action of hawthorn-probiotic postbiotics involves multiple targets including CASP8/CASP3-mediated cell death as well as STAT3/NF-κB signaling pathways.

**Conclusions:**

Postbiotic of hawthorn-probiotic demonstrates efficacy in alleviating constipation through the multi-pathway inhibition of apoptosis and pyroptosis, alongside the remodeling of the immune microenvironment. Its multi-target mechanism and favorable safety profile position it as a promising therapeutic candidate. These findings lay a solid foundation for the development of interventions based on hawthorn-probiotic postbiotics for constipation and related gastrointestinal disorders.

## 1 Introduction

Constipation, a globally prevalent gastrointestinal dysfunction disease, affects approximately 10-20% of adults, with particularly high rates observed in aging populations. This condition is experiencing a significant upward trend (1). In recent years, shifts in dietary habits and lifestyle modifications have contributed to a rising global incidence of constipation. Elderly people are at high risk for chronic constipation, prevalence is further elevated to 30-40% in the elderly population over 65 years of age (2). Such high prevalence rates pose significant challenges to public health systems. According to the Global Burden of Disease Study, the socioeconomic cost of constipation has increased by 45% compared to a decade ago, mainly due to medical expenses and productivity losses (3). Current drug treatments are often accompanied by side effects (such as electrolyte imbalance and drug dependence), while dietary intervention has attracted much attention due to its safety and sustainability. Patients suffering from chronic constipation may also be at risk of serious health problems such as colorectal cancer and cerebrovascular disease, further highlighting the importance of constipation prevention and treatment (4). This study used loperamide to construct a mouse model of constipation. Loperamide is a peripheral *μ*-opioid receptor agonist that induces constipation by specifically acting on receptors in the enteric nervous system within the intestines. Its mechanism of action includes inhibiting the release of acetylcholine, thereby suppressing propulsive peristalsis, prolonging intestinal transit time, and promoting the absorption of water and electrolytes, leading to dry and hard stools. This model is highly suitable for simulating human slow-transit constipation, as it replicates the core clinical features of reduced stool frequency, decreased stool moisture, and delayed intestinal motility (5). The use of mice in research has been validated because they share important physiological similarities with humans, particularly in terms of gastrointestinal function and the gut-brain axis. The loperamide-induced model in mice is a well-established, reproducible, and widely accepted preclinical model for screening therapeutic agents, making it a practical and relevant choice for this study (6).

PANoptosis, a comprehensive mode of cell death, incorporates core features of Pyroptosis, Apoptosis and/or Necroptosis (7). In the pathological process of intestinal inflammatory diseases, the synergistic activation of multiple modes of Programmed Cell Death (PCD) leads to excessive death of Intestinal Epithelial Cells (IECs), which in turn disrupts the intestinal barrier function. The intestinal epithelial barrier is composed of tight junction proteins (such as ZO-1, occludin) and complete epithelial cell layer, inappropriate apoptotic processes accelerate intestinal damage and disrupt intestinal barrier function, and its dysfunction is the core link in the occurrence and development of constipation (8). It was found that colon epithelial cell apoptosis was significantly up-regulated under constipation, which was manifested by increased expression of pro-apoptotic protein BAX and decreased expression of anti-apoptotic protein BCL-2 (9). This imbalance of apoptosis exacerbates barrier damage through two mechanisms: On the one hand, excessive epithelial cell shedding destroys the tightly connected structure and weakens the integrity of the barrier; On the other hand, DAMPs released by apoptotic cells activate innate immune cells (such as macrophages), trigger local inflammatory responses (10), and further promote the increase of intestinal mucosal permeability. Barrier damage leads to translocation of intestinal endotoxins (such as LPS) into the blood, activation of TLR4/NF-κB signaling pathway (11), and abnormal secretion of pro-inflammatory factors (such as IL-1β, TNF-α), forming a vicious cycle of “barrier destruction - flora imbalance - inflammation amplification” (12). Disturbance of the flora (e.g. decrease of beneficial bacteria, proliferation of pathogenic bacteria) and insufficient synthesis of metabolites (e.g. short-chain fatty acids) further inhibit the ability of intestinal epithelial repair. Animal experiments have shown that specific inhibition of intestinal epithelial anti-apoptotic genes can induce constipation phenotype, accompanied by intestinal motility disorders and intestinal nervous system dysfunction (13). These findings reveal the central role of apoptosis imbalance in constipation by regulating the inflammatory - microbiota - neural interaction network, and provide a theoretical basis for novel therapeutic strategies targeting apoptosis pathways.

Recent studies have shown that certain probiotics and their metabolites (epigenin) can inhibit intestinal epithelial cell apoptosis through multiple pathways. Extracellular polysaccharide secreted by L9 of *Lactobacillus plantarum* has been confirmed to restore the expression of tight junction protein ZO-1 (14). *Bifidobacteria*-derived epigeneons (such as peptidoglycan) effectively inhibit the mitochondrial translocation of BAX protein and reduce the level of apoptosis by activating the PI3K/Akt signaling pathway (15). Clinical trials have further found that *Lactobacillus plantarum HNU082* dietary intervention can significantly reduce colon epithelial cell apoptosis in patients with constipation, accompanied by increased butyrate synthesis and improved intestinal barrier function (as validated by decreased serum connexin concentration) (16). These findings suggest that probiotic therapy based on apoptosis regulation mechanisms may become a new strategy for constipation treatment. This study selected *Lactobacillus* as the probiotic strain for postbiotic fermentation. In addition to its remarkable effect on improving intestinal epithelial cell apoptosis, this strain also shows encouraging results in improving constipation, specifically by increasing the frequency of bowel movements. Furthermore, *Lactobacillus paracasei* demonstrates significant advantages in effectively restoring intestinal microbial balance and regulating key physiological pathways (17, 18).

This study investigates the anti-constipation mechanism of postbiotic of hawthorn-probiotic (HP) from the perspective of intestinal epithelial cell apoptosis regulation. By delineating HP’s role in modulating apoptotic pathways, this research will provide novel insights into its multi-target regulatory mechanisms, while offering a theoretical foundation for developing next-generation constipation therapies with enhanced efficacy and safety.

## 2 Materials and methods

### 2.1 Preparation of hawthorn aqueous extract and postbiotic

The preparation protocol is based on our previous research (19). Briefly, each liter of distilled water was supplemented with 10 g casein digest, 10 g beef extract, 4 g yeast extract, 2 g triammonium citrate, 5 g sodium acetate, 0.2 g magnesium sulfate, 0.05 g manganese sulfate, 2 g dipotassium phosphate, 20 g dextrose, and 1.08 g Tween 80. The mixture was adjusted to pH 5.7 ± 0.2, sterilized by autoclaving (121°C, 20 min), and inoculated with *Lactobacillus paracasei* (isolated from baby feces, identified again by colony PCR, the sequence was shown in **Supplementary Table S1**). The culture was incubated at 35°C with shaking (220 rpm) until reaching an OD of 0.8–1.2(1 × 10^9^ CFU). A subset of the culture was harvested, while the remainder underwent extended fermentation (72 h). Probiotics supernatant was obtained by centrifugation (12,000 × g, 10 min) and sterile filtration (0.22 μm membrane). For hawthorn aqueous extract, dried hawthorn was soaked in distilled water (30 min), boiled (30 min), concentrated to 1 g/mL, and filtered (0.22 μm membrane). Sterilized hawthorn extract was combined with the bacterial suspension and fermented under aerobic conditions (35°C, 220 rpm, 72 h). The final postbiotic was isolated via centrifugation (12,000 × g, 10 min) and filtration (0.22 μm membrane).

### 2.2 Animal experiments

Male KM mice (240-day-old) were purchased from Guangzhou Regal Biotechnology Co., Ltd. (SCXK [Yue] 2023-0059; SYXK [Yue] 2023-0182) and housed under controlled conditions (25 ± 2°C, 12 h light/dark cycle) with free access to food and water. All experimental protocols were approved by the Animal Ethics Committee of Guangzhou University of Traditional Chinese Medicine. Following a 1-week acclimatization, mice were randomly divided into five groups (n = 5): the normal control group (N) received daily distilled water gavage, while the remaining mice were administered loperamide (5 mg/kg/day) for 7 days to establish a constipation model. Subsequently, constipated mice were assigned to four groups: model group (M) (no intervention), hawthorn group (H) (0.2 mL/day hawthorn aqueous extract, 1 g/mL), probiotics group (P) (0.2mL *Lactobacillus paracasei* supernatant, equivalent to the metabolites/day), and hawthorn-probiotic probiotics group (HP) (0.2 mL/day hawthorn-probiotic postbiotic), with treatments continued for 7 days. Body weight and fecal water content were monitored throughout the study. Mice were sacrificed after cervical dislocation after inhalation of 5% isoflurane anesthesia. After euthanasia, colon tissues and the spleen were taken for further analysis.

### 2.3 Preparation of gut microenvironment-mimicking conditioned medium

To simulate the gut microenvironment *in vitro*, fresh intestinal contents were aseptically collected from the colonic tissues of KM mice, immediately snap-frozen in liquid nitrogen, and homogenized using a sterile mortar and pestle. The homogenate was resuspended in ice-cold RPMI-1640 medium (Gibco, USA) at a concentration of 100 mg/mL and subjected to continuous agitation (200 rpm, 1 h, 4°C) to ensure uniform dispersion of microbial and soluble components (20). The suspension was sequentially filtered through 0.45 μm (Millipore, USA) and 0.22 μm sterile syringe filters under laminar flow to remove particulate matter while retaining soluble microbial metabolites and bioactive factors. The resulting conditioned medium, representing a physiologically relevant gut microenvironment, was diluted 1:4 (v/v) with fresh RPMI-1640 prior to cell treatment.

### 2.4 Cell culture

NCM460 cells (National Collection of Authenticated Cell Cultures, China) were maintained in RPMI-1640 medium (Gibco, USA), CaCO2 cells (National Collection of Authenticated Cell Cultures, China) were maintained in DMEM medium (Gibco, USA). Cells were supplemented with 10% fetal bovine serum (Gibco, USA) and 1% penicillin-streptomycin (Gibco, USA) at 37°C under a humidified 5% CO2 atmosphere.

### 2.5 RNA isolation and quantitative analysis

Total RNA was extracted from colon tissues using FreeZol Reagent (Vazyme, China) according to the manufacturer’s instructions. cDNA synthesis and genomic DNA removal were performed with the HiScript IV 1st Strand cDNA Synthesis Kit (+gDNA wiper, Vazyme, China). Quantitative PCR was conducted using ChamQ SYBR qPCR Master Mix (Vazyme,China) in 20 μL reactions under the following conditions: 95°C for 1 min, followed by 40 cycles of 95°C (15 s) and 60°C (30 s). GAPDH served as the reference gene for normalization. Relative mRNA expression levels were calculated using the 2-ΔΔCt method, with all reactions performed in triplicate. Primer sequences are given in **Table 1**.

**Table 1:** Primer sequences for qRT–PCR.

Primer name	Forward	Reverse
*H-GSDMD*	5’-CCAGAAGAAGACGGTCACCATCC-3’	5’-ACAACACCAGGCACTCCAGGA-3’
*H-caspase1*	5’-TCTCACTGCTTCGGACATGACTACA-3’	5’-CCACATCACAGGAACAGGCATATTCT-3’
*H-caspase3*	5’-GGATGGCTCCTGGTTCATCC-3’	5’-TCTGTTGCCACCTTTCGGTT-3’
*H-caspase9*	5’-GACCAGAGATTCGCAAACCAGAGG-3’	5’-AAGAGCACCGACATCACCAAATCC -3’
*H-TNFR1*	5’-ACCACCTCAGACACTGCCTCAG-3’	5’-CGGTCCACTGTGCAAGAAGAGATC-3’
*H-TNF-α*	5’-TGGCGTGGAGCTGAGAGATAACC-3’	5’-GACGGCGATGCGGCTGATG-3’
*H-ZBP1*	5’-GCTCAACCAAGTCCTCTACCGAATG-3’	5’-TGTCCAGAAGGTGCCTGCTCTT-3’
*H-GAPDH*	5’-GTGGACCTGACCTGCCGTCTAG-3’	5’-GAGTGGGTGTCGCTGTTGAAGTC-3’
*M-caspase3*	5’-GACTGGAAAGCCGAAACTCTTCATC-3’	5’-ATGAGAGAGGATGACCACCACAAAG-3’
*M-GSDMD*	5’-AGTCCCACTGTCTGTCTCAATGC-3’	5’-CGATGGCATGGTCCTCGATT-3’
*M-ZBP1*	5’-CAGCCATTCTTGCCTGTGGA-3’	5’-TGCTCCAGATTGTCTCCTGTG-3’
*M-GAPDH*	5’-AATGGTGAAGGTCGGTGTGA-3’	5’-CGCTCCTGGAAGATGGTGAT-3’

### 2.6 Western blot

Approximately 200 mg of colon tissue or cell samples were homogenized in 1 mL RIPA lysis buffer (Servicebio, China) containing 1 mM PMSF protease inhibitor. Lysates were incubated at 4°C for 30 min, centrifuged (12,000 × g, 15 min), and supernatants were collected. Protein concentrations were determined using a BCA assay kit (Beyotime, China). Equal protein amounts were separated by SDS-PAGE and transferred to PVDF membranes (Millipore, USA). Membranes were blocked with 5% non-fat milk for 1 h, incubated overnight at 4°C with primary antibodies Caspase 1 (1:1000, M025280, Abmart), NLRP3(1:1000, P60622R3, Abmart), GSDMD(1:1000, PU224937, Abmart), Caspase3(1:500, GTX110543, GeneTex), Caspase9(1:1000, 9508S, CST), BAX(1:1000, WL01637, Wanleibio), Bcl-2(1:1000, CY6717, Abways), β-actin(1:3000, GB15003-100, Servicebio), and subsequently with HRP-conjugated secondary antibodies (1:10000, GB23303, Servicebio) for 1 hour at room temperature. Protein signals were visualized using a chemiluminescence imaging system (Tanon, China), and band intensities were quantified with ImageJ v1.48 (NIH, USA).

### 2.7 Fluorescence microscopy detection

Cells were seeded in culture dishes according to experimental design. After treatment, the medium was aspirated, and cells were washed once with PBS before adding YP1/PI working solution (Beyotime, China). Following a 15-min incubation in the dark, fluorescence was observed under an inverted microscope with excitation/emission wavelengths of 491/509 nm (YP1, green) and 535/617 nm (PI, red).

### 2.8 Cell viability assay

Cells (8,000/well) were seeded in 96-well plates. After adherence, fresh conditioned medium containing 10 μg/mL LPS (Sigma, USA) or gut microenvironment-mimicking conditioned medium (H, P, HP) was added. At 24 h, 10 μL CCK-8 reagent (NCM Biotech, China) was introduced, and absorbance at 450 nm was measured after 2 hours incubation using a multimode microplate reade.

### 2.9 Flow cytometry

Cells treated for 24 h were harvested by centrifugation (300 × g, 5 min), washed with PBS, and resuspended in 500 μL 1×Buffer. Add Annexin V-FITC/PI (5 μL each, Elabscience, China) or YO-PRO-1/PI (0.5ul each, beyotime,China), mix gently, and incubate for 15–20 min at room temperature in the dark. The apoptosis rate was analyzed using a flow cytometer (BD Accuri C5, USA).

### 2.10 Identification of major components in the postbiotic of hawthorn-probiotic

The sample pretreatment process is as follows: Take 100 μL of postbiotic liquid sample, add 400 μL of pre-chilled methanol, vortex mix, then incubate at -20°C for 30 minutes to precipitate proteins. Centrifuge at 20,000 rcf at 4°C for 15 minutes, transfer 400 μL of the supernatant to a new tube, centrifuge again, and transfer the supernatant to a sample vial for analysis. All operations are performed on ice. Concurrently, equal volumes of each sample are mixed to prepare quality control (QC) samples for system stability monitoring.

Chromatographic separation is performed using a Thermo Vanquish Flex UPLC system equipped with an ACQUITY UPLC T3 column (100 mm × 2.1 mm, 1.8 μm), with a column temperature of 40°C and a flow rate of 0.35 mL/min. Mobile phase A is an aqueous solution containing 5 mmol/L ammonium acetate and 5 mmol/L acetic acid, and phase B is acetonitrile. The gradient elution program is as follows: 0–20 min, phase B increases from 5% to 70%; 22–25 min, phase B increases to 95% and remains constant; 25.1–30 min, phase B decreases to 5% for column equilibration.

Mass spectrometry detection was performed using an Orbitrap Exploris 120 mass spectrometer, with data collected in both positive and negative ion modes. Ion source parameters were set as follows: curtain gas pressure 1, auxiliary gas pressure 12, sheath gas pressure 50, ion source temperature 350°C; positive ion mode spray voltage +3800 V, negative ion mode –3400 V. Data-dependent acquisition (DDA) mode was used: primary scan range m/z 70–1050, resolution 60,000; the top 4 ions with a selected intensity exceeding 5000 were selected for secondary fragmentation, with a secondary resolution of 15,000 and a dynamic exclusion time set to 6 s. A QC sample was inserted every 10 samples to monitor and correct system errors.

### 2.11 Network pharmacology analysis

Potential targets of HP were predicted using the SwissTargetPrediction and SuperPred databases, followed by standardization in UniProt. Constipation-related targets were identified from OMIM and GeneCards using “constipation” as a keyword, while PANoptosis-related genes were obtained by searching “pyroptosis,” “apoptosis,” and “necroptosis” in GeneCards, yielding 261 unique genes after deduplication (21). These three gene sets—HP targets, constipation-related targets, and PANoptosis genes—were intersected using Wei Sheng Xin to identify overlapping targets, representing potential mechanisms for HP in treating constipation via PANoptosis modulation. The overlapping targets were used to construct a protein-protein interaction (PPI) network in STRING with a confidence score ≥0.9. The network was analyzed and visualized using Cytoscape. Functional enrichment analysis of the core targets was performed in DAVID, including Gene Ontology (GO) and Kyoto Encyclopedia of Genes and Genomes (KEGG) pathway analyses, to elucidate the biological processes and signaling pathways involved in HP’s effects (URLs of database are shown in **Supplementary Table S2**) (22).

### 2.12 Statistical analysis

The statistical analyses and graphical representations of the experimental data were expressed as “mean ± standard deviation” and performed using GraphPad Prism 8.0 (GraphPad Inc., San Diego, CA, USA) and IBM SPSS Statistics 22.0 (IBM Corp., Armonk, NY, USA). Levene's test was employed to assess homogeneity of variance. If the test indicated significant variance (p < 0.05), further comparisons between groups were conducted using ordinary one-way ANOVA. If variance was found to be heterogeneous (p ≥ 0.05), subsequent analysis was performed using the Kruskal-Wallis nonparametric test. Statistical significance was set at P < 0.05.

## 3 Results

### 3.1 Postbiotic of hawthorn-probiotic protect the intestinal barrier by altering the intestinal microenvironment to mitigate PANoptosis of intestinal epithelial cells

**Figure 1:**
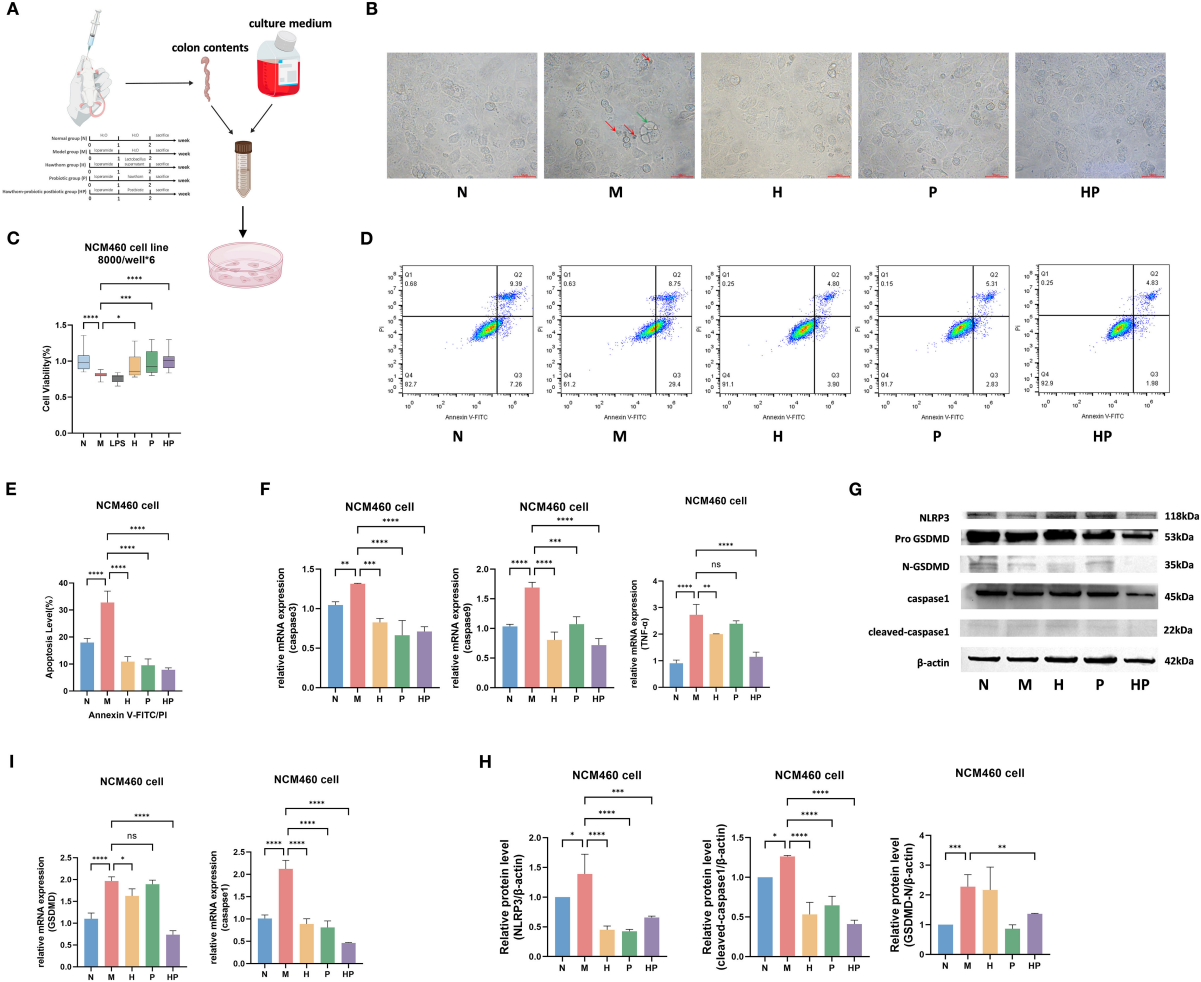
Postbiotic of hawthorn-probiotic can alleviate apoptosis and pyroptosis in intestinal epithelial cells by altering the intestinal microenvironment. **(A)** Animal experiments and preparation of media simulating the intestinal microenvironment. The pattern was created with BioGDP.com. (23) **(B)** Cell status in different groups of intestinal microenvironmental media. The red arrow points to apoptosis. The green arrow points to pyroptosis. **(C)** Cell viability of NCM460 cells cultured in different groups of intestinal microenvironment medium for 24 hours was detected by CCK8. **(D, E)** NCM460 cells relative apoptosis level (Q2+Q3). **(F)** The mRNA expression levels of apoptosis-related genes. **(G, H)** The protein levels of pyroptosis-related genes. **(I)** The mRNA expression levels of pyroptosis-related genes. N, conditioned medium of the normal group. M, conditioned medium of the model group H, conditioned medium of the hawthorn group P, conditioned medium of the probiotics group HP, conditioned medium of the postbiotic of hawthorn-probiotic group. NS; *P<0.05; **P<0.01; ***p<0.001, ****p<0.0001.

As shown in the previous study, the model group (M) mice exhibited typical constipation symptoms (decreased frequency of defecation, dry feces, etc.), whereas the three drug groups, hawthorn (H), probiotics (P), postbiotic of hawthorn-probiotic (HP) all significantly improved these symptoms, with the most significant effect in the HP group (19) To further explore the mechanism of action of HP, we prepared conditioned medium with intestinal contents to mimic the intestinal microenvironment of different groups of mice for the cultivation of normal colonic epithelial cells NCM460 (**Figure 1A**). Microscopic observations revealed increased cell death and abnormal morphology in the conditioned medium simulating the intestinal microenvironment of the group M, and the presence of apoptosis and pyroptosis was observed. Apoptotic cells appeared compressed in size and nuclei(red arrows), and pyroptotic cells were swollen and expanded with multiple bubble-like projections (green arrow) (24). In contrast, the cell status in the conditioned medium simulating the intestinal microenvironment of drug group (especially the HP group) significantly improved, the cell morphology was close to that of the N group (**Figure 1B**). The 24-hour cell viability was detected by CCK-8 assay, compared with the normal group (N, 100.0 ± 3.2%), the cell viability was significantly decreased in the model group (M) (81.3 ± 4.1%, ***p<0.001). Meanwhile, cells treated with 10ug/ml LPS, a potent inflammatory stimulant, served as a positive control for inflammation-induced cell damage and, as expected, also showed a significant decrease in cell viability. This result may indicate that the conditioned medium of the model group contains inflammatory factors that cause cell damage equivalent to that caused by 10μg/ml LPS. Among the drug intervention groups, H (92.5 ± 6.3%), P (99.2 ± 7.2%) and HP (100.6 ± 5.9%) groups significantly improved cell viability (***p<0.001 vs. M), with the most significant effect in HP group, which was completely restored to the normal level (p>0.05 vs. N) (**Figure 1C**). These results confirmed that the drug intervention groups were all effective in reversing the cellular damage induced by the inflammatory milieu, with HP having the most significant protective effect.

#### 3.1.1 Inhibition of CASP3-mediated apoptosis via microenvironmental modulation

Annexin V-FITC/PI double-staining flow analysis showed that the apoptosis rate of cells of the M group was 32.77%(Q2+Q3), which was significantly higher than the cell of the N group (17.96%, ***p < 0.001). Three groups of therapeutic interventions showed significant anti-apoptotic effects: the H group exhibited 10.88% apoptosis, the P group 9.55%, and the HP group 7.89% - all significantly lower than the M group (p < 0.001). Notably, the HP group produced the most robust reduction (**Figures 1D, E**). During apoptosis, Caspase-3, Caspase-9 and TNF-α together constitute the core regulatory network of the apoptosis signaling pathway, acting as terminal effector execution molecules, mitochondrial pathway initiation activators and death receptor-mediated exogenous signaling hubs, respectively. We examined the mRNA expression levels of these genes and found that the mRNA levels of apoptosis-related genes were significantly increased in the M group and decreased in the H, P and HP groups (**Figure 1F**).

#### 3.1.2 Blockade of NLRP3-GSDMD pyroptosis by microenvironment remodeling

In pyroptosis, NLRP3 initiates inflammatory vesicle assembly by recognizing exogenous and exogenous signals; Caspase-1 shears Gasdermin D and catalyzes IL-1β/IL-18 maturation; and the formation of membrane pores by GSDMD mediates cell rupture and inflammatory mediator release. We examined the mRNA and protein expression levels of these genes, and found that the expression levels of the genes related to pyroptosis were significantly higher in group M and lower in groups H, P and HP. (**Figures 1G–I**).

#### 3.1.3 Attenuation of ZBP1-driven necroptosis through microenvironmental regulation

**Figure 2:**
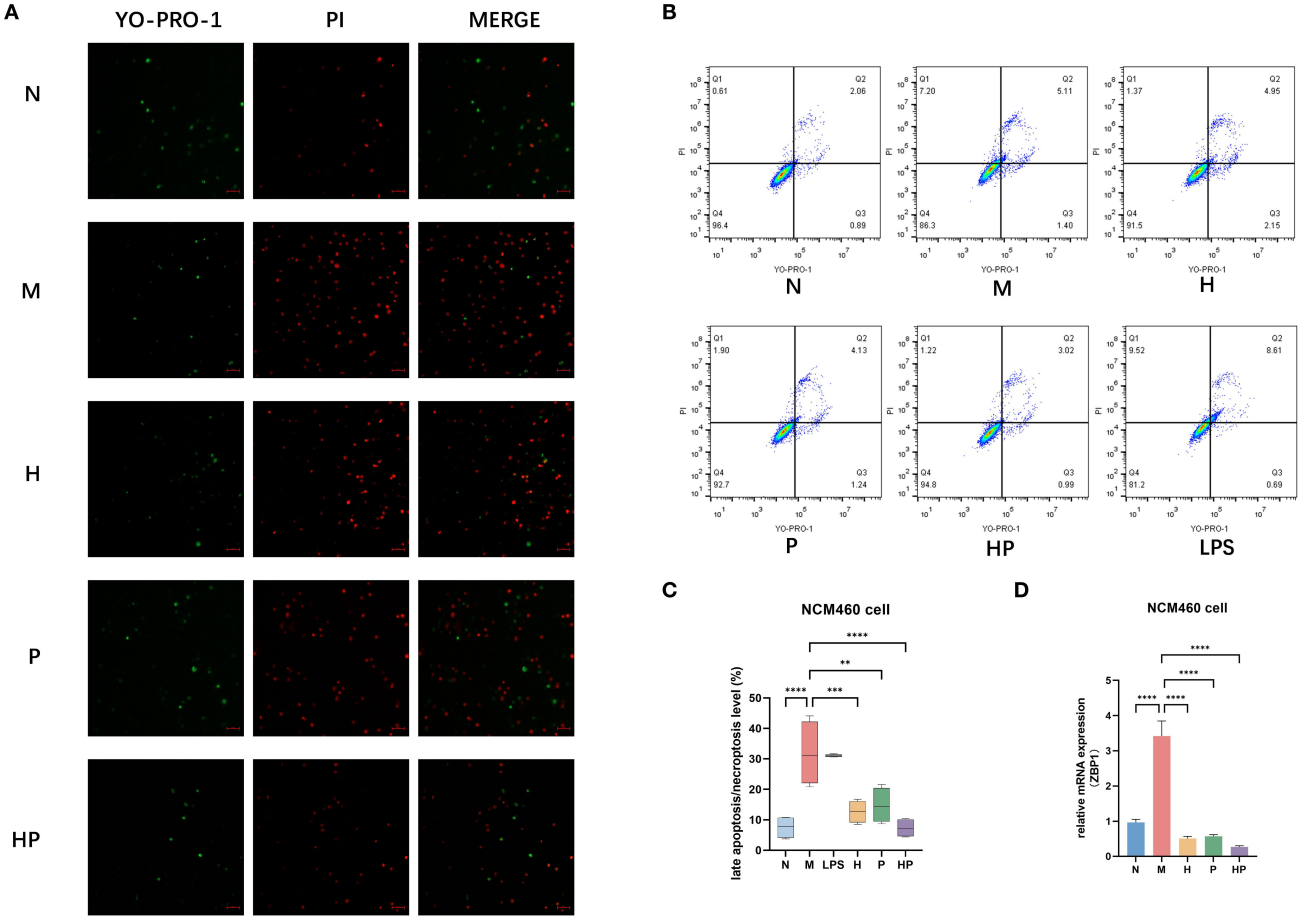
Postbiotic of hawthorn-probiotic can alleviate necroptosis in intestinal epithelial cells by altering the intestinal microenvironment. **(A)** YO-PRO-1/PI staining of each group of cells under fluorescence microscope. **(B, C)** NCM460 cells relative necroptosis level (Q1+Q2). **(D)** The mRNA expression levels of ZBP1. N: conditioned medium of the normal group. M: conditioned medium of the model group H: conditioned medium of the hawthorn group P: conditioned medium of the probiotics group HP: conditioned medium of the postbiotic of hawthorn-probiotic group. **p<0.01, ***p<0.001, ****p<0.0001.

Necroptosis was detected by YO-PRO-1/PI staining in each group. Fluorescence microscopy showed an increase in the number of both YO-PRO-1-positive (green fluorescence) and PI-positive (red fluorescence) cells in the M group compared with the N group, while the number of double-staining-positive cells was significantly reduced in the HP group (**Figure 2A**). Flow cytometry further confirmed these findings, and the trend of each group was consistent with the above results. Q4 represents the rate of viable cells; Q1 and Q2 represent the rate of late apoptosis or necrosis; Q3 represents the rate of early apoptosis. of early apoptosis. The necroptosis level in the HP group was 2.76%, which was significantly lower than that in the model group (5.25%) (** p < 0.01) (**Figures 2B, C**) (25). Since ZBP1 (Z-DNA binding protein 1) is a key regulatory molecule in the necrotic apoptotic pathway. We examined the mRNA expression level of ZBP1 and found that it was significantly elevated in the M group and significantly decreased in the H, P and HP groups, with the lowest in the HP group (**Figure 2D**).

### 3.2 Postbiotic of hawthorn-probiotic alleviate constipation in mice by regulating PANoptosis gene expression

**Figure 3:**
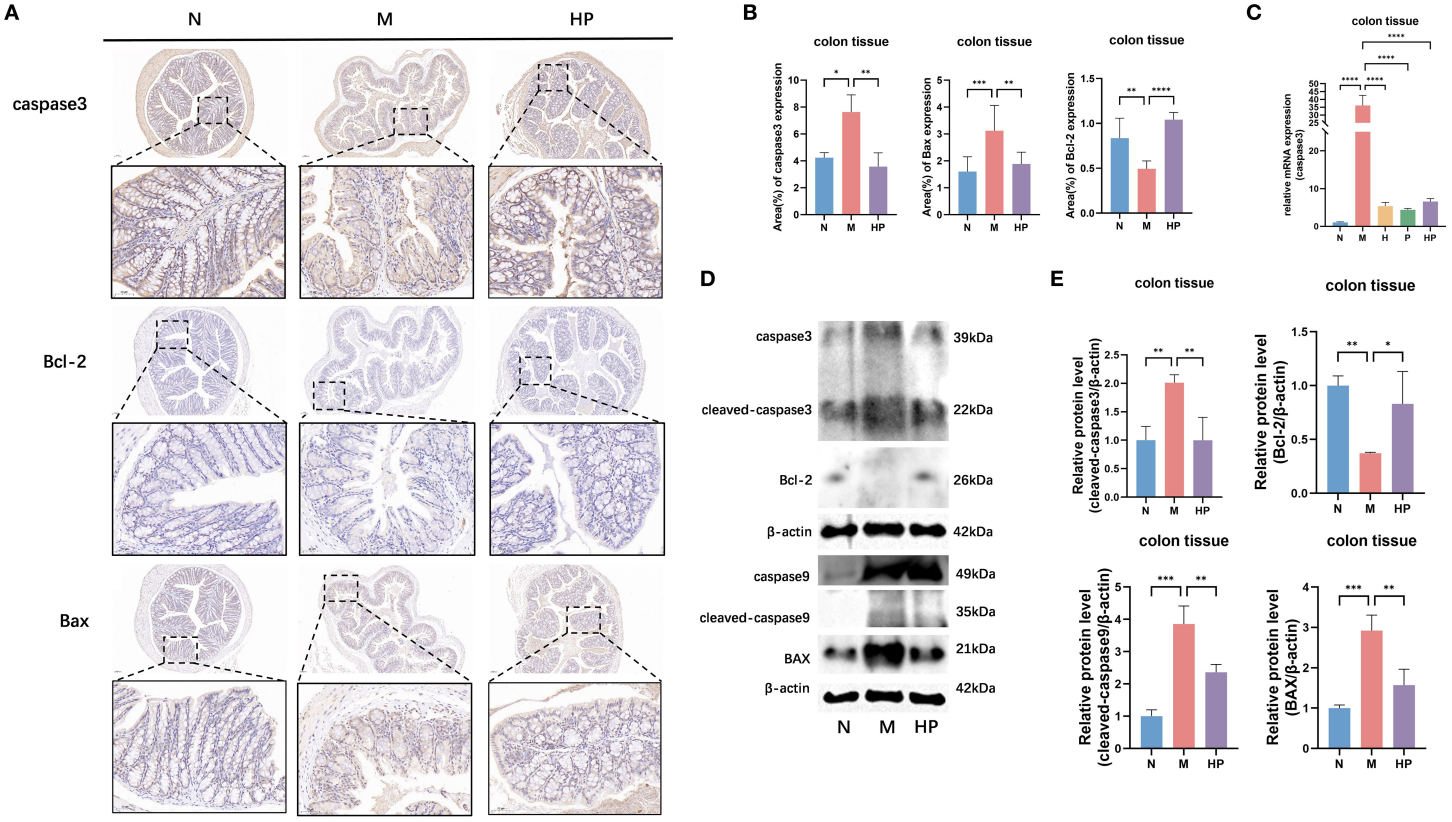
Postbiotic of hawthorn-probiotic alleviates constipation-induced apoptosis in the mouse colon. **(A, B)** Immunohistochemical analysis of caspase3, Bcl-2 and BAX in mouse colon tissues and statistics on the percentage of positive area. **(C, D)** The protein levels of apoptosis-related genes. **(E)** The mRNA expression levels of caspase3. N: normal group (mice gavaged with distilled water). M: model group (mice gavaged with loperamide). H: hawthorn group (mice gavaged with hawthorn aqueous extract). P: probiotics group (mice gavaged with *Lactobacillus paracasei*). HP: treatment group (mouse gavaged with the postbiotic of hawthorn-probiotic). *p<0.05, **p<0.01, ***p<0.001, ****p<0.0001.

IHC and Western blotting results of colon tissues from group M mice showed increased protein levels of apoptosis-related genes (caspase3, BAX, Bcl-2 and caspase9) (**Figures 3A–D**), and qPCR results also showed a significant increase in the mRNA level of caspase3 in colon tissues from group M mice (**Figure 3E**). The protein levels and mRNA levels of apoptosis-related genes in the colonic tissues of mice in the HP group were reduced compared with those in the M group. These results suggest that HP mitigates colonic tissue damage by blocking CASP3-mediated apoptosis via Bcl-2/Bax axis modulation.

**Figure 4:**
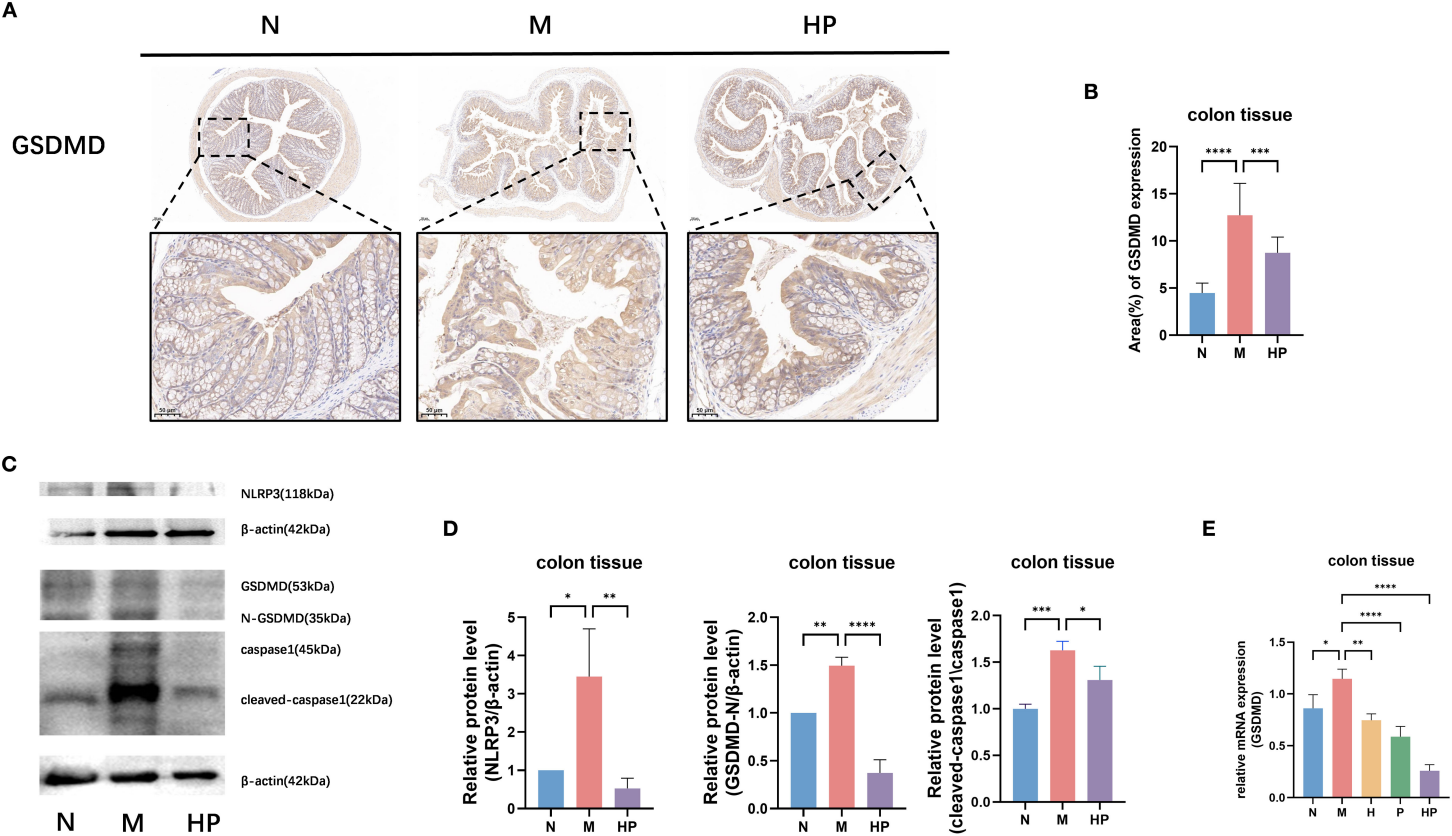
Postbiotic of hawthorn-probiotic alleviates constipation-induced pyroptosis in the mouse colon. **(A, B)** Immunohistochemical analysis of GSDMD in mouse colon tissues and statistics on the percentage of positive area. **(C, D)** The protein levels of pyroptosis-related genes. **(E)** The mRNA expression levels of GSDMD. N: normal group (mice gavaged with distilled water). M: model group (mice gavaged with loperamide). H: hawthorn group (mice gavaged with hawthorn aqueous extract). P: probiotics group (mice gavaged with *Lactobacillus paracasei*). HP: treatment group (mouse gavaged with the postbiotic of hawthorn-probiotic). *p<0.05, **p<0.01, ***p<0.001, ****p<0.0001.

The results of IHC and Western blotting showed that the protein level expression of pyroptosis protein GSDMD in the colon tissue of mice in the M group was increased compared with that in the N group. Meanwhile, the results in the HP group were decreased compared with those in the M group (**Figures 4A–D**). The qPCR results were consistent with the above results (**Figure 4E**).

**Figure 5:**
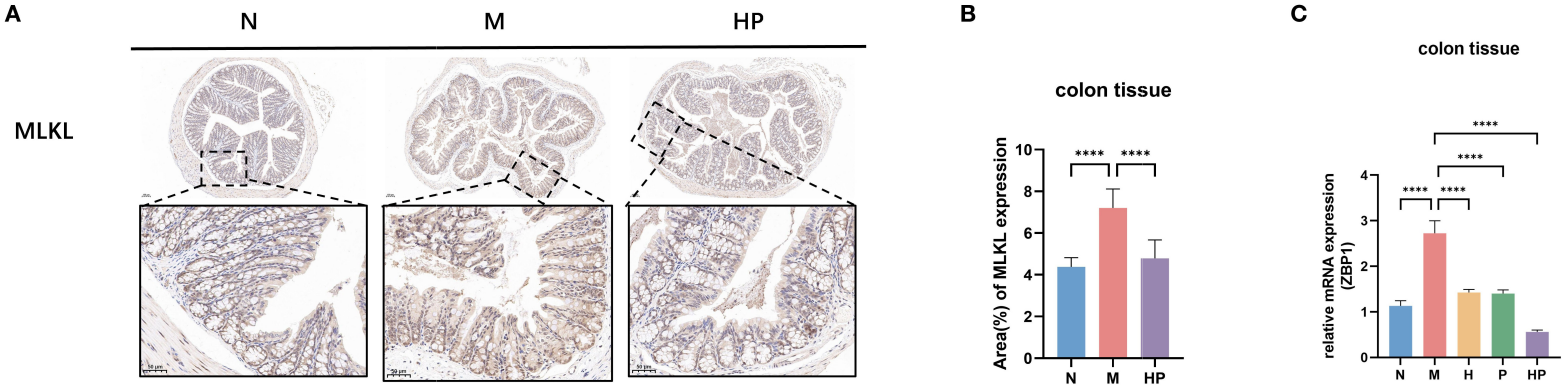
Postbiotic of hawthorn-probiotic alleviates constipation-induced necroptosis in the mouse colon. **(A, B)** Immunohistochemical analysis of MLKL in mouse colon tissues and statistics on the percentage of positive area. **(C)** The mRNA expression levels of ZBP1. N: normal group (mice gavaged with distilled water). M: model group (mice gavaged with loperamide). H: hawthorn group (mice gavaged with hawthorn aqueous extract). P: probiotics group (mice gavaged with *Lactobacillus paracasei*). HP: treatment group (mouse gavaged with the postbiotic of hawthorn-probiotic). ****p<0.0001.

The protein level and mRNA level of necroptosis genes were detected by IHC and qPCR, respectively. The results showed that the protein level expression of MLKL in the colon tissue of mice in group M increased, and it could be improved in group HP (**Figures 5A, B**). The mRNA level of ZBP1 in the colon tissue of mice in group M increased, but was reversed in group HP (**Figure 5C**).

### 3.3 Postbiotic of hawthorn-probiotic alleviates constipation and reshapes intestinal homeostasis by regulating the balance between inflammation and defense

**Figure 6:**
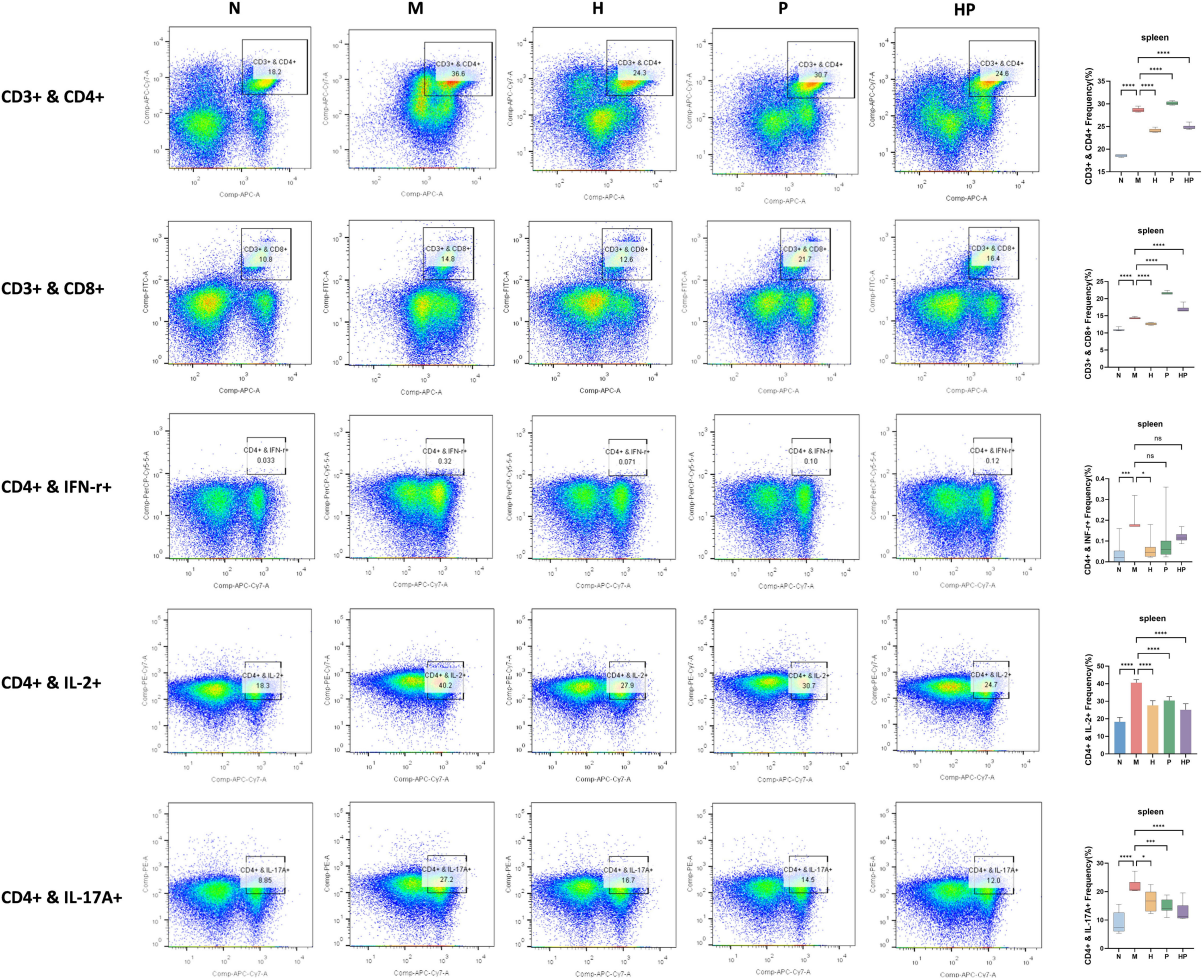
Characterization of T-lymphocyte subpopulations and cytokine secretion in the mouse spleen. Frequency of CD3+ & CD4+, CD3+ & CD8+, CD4+ & IFN-r+, CD4+ & IL-2+, CD4+ & IL-17A+ in the spleens of each group of mice using flow cytometry. NS; *p<0.05, ***p<0.001, ****p<0.0001.

In the previous study, we found that the white medulla of the spleen in group M mice was blurred and the distribution of immune cells was reduced. The above lesions were significantly alleviated in group HP. Therefore, we further analyze the proportion of spleen lymphocyte subgroup. The proportion of CD3+CD4+ T cells in the spleen of mice in group M was significantly increased, suggesting excessive activation of helper T cells under constipation. The proportion of this subgroup in group HP was significantly adjusted back, indicating that it can block the inflammatory cascade reaction by inhibiting the abnormal proliferation of CD4+ T cells. The proportion of IL-2+ CD4+ T cells in group M reached 40.2%. This excessive activation might suggest that in constipation, the spleen exacerbates the hyperactivity of intestinal mucosal immune response by promoting B cell differentiation. However, after HP intervention, this proportion dropped sharply to 24.7%, directly confirming its inhibitory effect on the humoral immune pathway. The abnormal expansion of IL-17A+ CD4+ T cells in group M constitutes another key pathological link. The IL-17 secreted by them can induce neutrophil infiltration and intestinal barrier dysfunction. The significant decrease after HP intervention indicates that it can effectively block this pro-inflammatory axis. It is worth noting that although Th1-type IFN-γ+ CD4+ T cells showed a slight decrease of 0.12% in the model group, the HP group maintained a basal level of 0.32%. This non-statistically significant change precisely reflects the immunomodulatory wisdom of the preparation - inhibiting excessive inflammation while retaining the IFN-γ -mediated antiviral defense function. The IL-2+ subset in CD8+ T cells of group M expanded to 14.9%. This enhanced cytokine secretion may aggravate immunopathological damage. Although it slightly decreased after HP intervention, it was not statistically significant, suggesting that it balanced the cytotoxic effect and tissue protection by moderately inhibiting IL-2. In contrast, there was no significant difference in the proportion of IFN-γ+ CD8+ T cells among the groups. This selective regulation ensured that the direct killing function of cytotoxic T cells was not inhibited, providing an immune barrier for eliminating potential intestinal pathogens. This bidirectional regulatory model, which both inhibits excessive inflammation and retains necessary defenses, ultimately constructs a brand-new regulatory paradigm of intestinal immune homeostasis by reshaping the Th1/Th2/Th17 balance and precisely regulating the function of CD8+ T cells (**Figure 6**; **Supplementary Figure S1**).

### 3.4 Network pharmacology analysis

**Figure 7:**
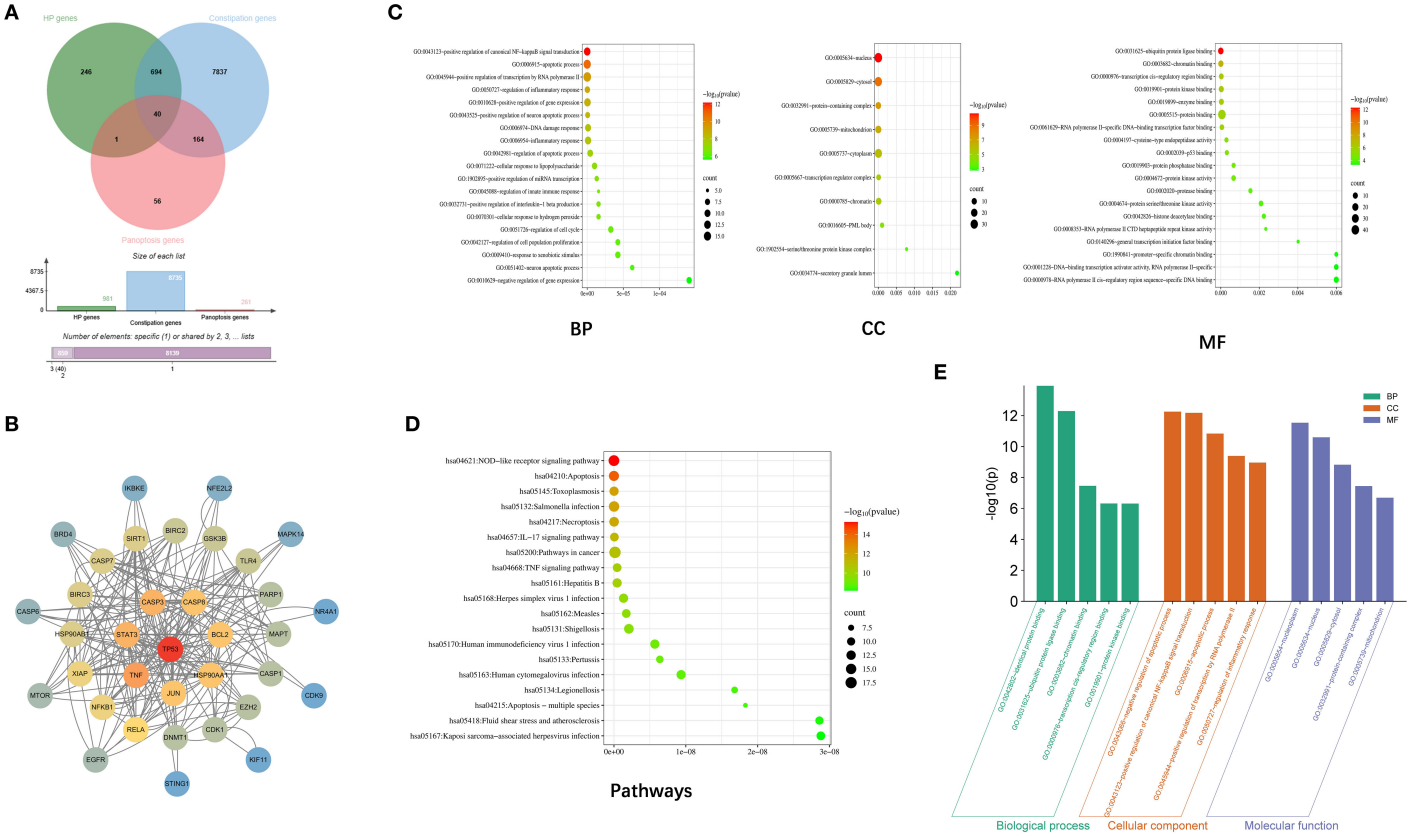
Network pharmacology analysis. **(A)** Intersection of Panoptosis-related genes, constipation relaed genes and postbiotic of hawthorn-probiotic related genes. **(B)** Construction of protein-protein interaction networks. Colors represent degree from high (red) to low (blue). **(C-E)** GO, KEGG enrichment analysis. Bargraph for DEGs in top 20 GO terms analysis. Each column corresponds to a term. the higher the column, the smaller P.

To further investigate the mechanism by which postbiotic of hawthorn-probiotic(HP) treats constipation by alleviating Panoptosis, we performed network pharmacological analyses. Based on the intersecting genes, we observed 40 significant PANoptosis-related genes in HP treatment of constipation (gene names were shown in **Supplementary Table S3**), then construct the Construction of protein-protein interaction networks (**Figures 7A, B**). The top 10 core targets identified from the PPI network were TP53, TNF, BCL2, STAT3, JUN, CASP3, CASP8, RELA, HSP90AA1, and NFκB1, which are closely associated with apoptosis, inflammation, and cellular stress responses. We observed a significant enrichment of multiple GO terms, including positive regulation of canonical NF-kappaB signal transduction, apoptotic process, positive regulation of transcription by RNA polymerase II, nucleus, cytosol, protein-containing complex, ubiquitin protein ligase binding (**Figure 7C**). KEGG pathway, which displayed NOD-like receptor signaling pathway, IL-17 signaling pathway, TNF signaling pathway, which plays an important role in inflammatory responses, immune defense, and the occurrence and development of various diseases (**Figure 7D**).

## 4 Discussion

Although this study illustrates the mechanism by which hawthorn probiotic postbiotics (HP) alleviate constipation by regulating the intestinal microenvironment, thereby inhibiting PANoptosis of intestinal epithelial cells, there are still several limitations that need to be further analyzed in subsequent studies.

Constipation has long been regarded as a functional disorder, but recent studies have shown that low-grade inflammation and intestinal epithelial barrier damage play a key role in its pathogenesis (26, 27). This study focuses on three executor proteins of programmed cell death pathways: Caspase-3 (CASP3), Gasdermin D (GSDMD), and MLKL. These proteins represent the terminal effector molecules of apoptosis, pyroptosis, and necrotic apoptosis, respectively, and their activation is a direct cause of epithelial barrier disruption. CASP3, as a key executor of apoptosis, its activation leads to DNA fragmentation and loss of phospholipid asymmetry in the cell membrane (28). In a state of constipation, its upregulation suggests sustained epithelial cell loss and impaired barrier integrity, consistent with previous reports of increased colonic mucosal apoptosis in constipated patients (29). Following HP treatment, CASP3 mRNA and protein levels were significantly reduced, and the mitochondrial apoptosis pathway was inhibited by regulating the Bcl-2/Bax balance, indicating that HP can promote the maintenance and repair of epithelial structure by suppressing excessive apoptosis, which is crucial for preventing chronic barrier dysfunction. In addition, HP treatment significantly reduced the mRNA expression of TNF-α and caspase-3 in Caco-2 and NCM460 intestinal epithelial cells after LPS stimulation (**Supplementary Figure S2**), indicating that HP contains bioactive compounds that can directly regulate host cell signaling to inhibit inflammatory and cell death pathways. This suggests that HP may exert its effects through both direct and indirect mechanisms. Pyroptosis is a pro-inflammatory form of programmed cell death mediated by GSDMD-induced formation of membrane pores (30). This study found that GSDMD expression was significantly elevated in the constipation model group, consistent with the inflammatory state of the intestine associated with constipation (31). It is known that GSDMD activation leads to the release of potent inflammatory factors such as IL-1β and IL-18 (32), further recruiting immune cells and amplifying the inflammatory response, forming a vicious cycle. Following HP intervention, GSDMD protein and mRNA levels decreased, indicating that it can effectively block this pro-inflammatory-apoptotic axis, not only reducing epithelial cell lysis but also potentially lowering local inflammatory burden, thereby preventing disease chronicity. Necrotic apoptosis is typically activated when other death pathways are blocked, with MLKL oligomerization and membrane translocation serving as hallmarks (33). This study shows that MLKL and its upstream regulatory molecule ZBP1 expression increases in the constipation model, suggesting a potential backup death mechanism triggered by microenvironmental stress, such as inflammation or cytokine stimulation. Previous studies have suggested that necrotic apoptosis is involved in various chronic inflammatory bowel diseases (34, 35). HP reduces the expression of MLKL and ZBP1, indicating that it can broadly inhibit multiple death pathways, avoiding compensatory activation caused by the inhibition of a single pathway (36), thereby providing more comprehensive protection of the epithelial barrier and having important preventive significance for maintaining long-term intestinal stability. Recent studies have indicated that PANoptosis plays a crucial role in the pathogenesis of inflammatory bowel disease (37). The downregulation of CASP3, GSDMD, and MLKL expression observed after HP treatment suggests that its protective effects on epithelial barrier integrity and anti-inflammatory properties may have broader therapeutic applications beyond constipation. However, further studies with specialized designs are needed to validate the efficacy of HP in other inflammatory bowel diseases. Although this study confirmed the protective effect of HP by simulating the intestinal microenvironment through culture supernatant, further confirmation of the cellular targets of HP’s direct action (such as immune cells and epithelial cells) is needed through co-culture experiments or *in vivo* cell-specific knockout models.

This study also investigated changes in splenic T lymphocyte subsets, with these immune markers reflecting systemic and mucosal immune imbalance in constipation. Abnormal proliferation of IL-17A+ CD4+ T (Th17) cells is a core feature of constipation-related inflammation. Th17 cells secrete large amounts of IL-17, which recruits neutrophils and disrupt epithelial tight junctions, consistent with reported immune cell infiltration and impaired barrier function in the colonic mucosa of constipated patients (38). HP significantly suppresses the Th17 response, directly alleviating the damaging effects of its primary effectors on the intestinal barrier, thereby fundamentally alleviating inflammatory damage. Concurrently, HP treatment preserves the function of IFN-γ+ Th1 and CD8+ T cells. This selective immune regulatory pattern holds significant disease prevention implications: on one hand, it suppresses pathogenic Th17 responses, while on the other hand, it maintains Th1/CTL-mediated immune surveillance against intracellular pathogens (39), thereby avoiding the increased infection risk associated with traditional immunosuppression and contributing to the long-term stability of immune balance. However, it should be noted that this study primarily evaluated systemic immune responses in the spleen. Since immune responses in the intestinal tract are also critical for constipation, whether HP regulates intestinal T cell populations remains to be further explored in future studies on intestinal mucosa-associated lymphoid tissue.

In this study, network pharmacology analysis provided valuable mechanistic hypotheses for future research. The top predicted targets TP53, TNF, and STAT3 were highly correlated with the core pathophysiology observed in our model. TP53 is a key regulator of cell cycle arrest and apoptosis (40). The predicted inhibitory effect of HP on TP53 aligns with the reduced epithelial cell apoptosis observed in our experiments, potentially explaining the underlying mechanism by which HP enhances intestinal barrier integrity. TNF is a major pro-inflammatory cytokine that can directly impair intestinal motility and promote inflammatory cell death (41). Targeting the TNF signaling pathway may explain the potent anti-inflammatory effects of HP observed in this study. STAT3 is a key signaling node integrating multiple cytokine and growth factor signals, influencing inflammation and cell survival (42). This pathway may explain HP’s broad immunomodulatory and cell-protective effects. However, these findings remain within the realm of prediction, and these specific targets require validation through *in vitro* and *in vivo* experiments. Further research is needed to functionally validate the involvement of these signaling pathways predicted by KEGG analysis through the application of gene silencing techniques, such as siRNA or CRISPR-Cas9 knockout, or the use of pathway inhibitors.

This study reveals a new mechanism by which HP alleviates constipation from the perspective of proteins and immune markers. HP achieves this by synergistically inhibiting the three key death execution proteins CASP3, GSDMD, and MLKL, thereby completely blocking pan-optosis and protecting epithelial barrier integrity. Concurrently, it precisely regulates the Th17/Th1 balance to alleviate inflammation while preserving defensive functions, thereby reestablishing immune homeostasis. These findings provide a theoretical basis for the application of HP in preventive strategies for constipation and related functional gastrointestinal disorders.

## 5 Conclusion

This study deeply analyzed the multi-level mechanism of action of Postbiotic of hawthorn-probiotic (HP) in alleviating constipation and regulating intestinal homeostasis, revealing its systematic strategy of achieving intestinal protection by inhibiting the pan-optotic pathway, reshaping the immune balance and regulating the gene expression network. Studies have confirmed that HP significantly improves constipation symptoms by reconstructing the intestinal microenvironment. Its effect is not only reflected in the increase of the survival rate of intestinal epithelial cells, Furthermore, by blocking CASP3/CASP9-mediated apoptosis, inhibiting pyroptosis driven by the NLRP3-GSDMD axis, and regulating necrotic apoptosis in the ZBP1-MLKL pathway, a triple inhibitory effect on the pan-optotic pathway is formed. This protective effect is manifested at the tissue level as the specific down-regulation of the expression of key executive molecules such as CASP3, GSDMD and MLKL in colon tissue, confirming the complete protective chain of HP from cell death regulation to tissue repair. In the dimension of immune regulation, HP demonstrates precise immune regulatory capabilities: By inhibiting the excessive activation of Th2/Th17 cells, the hyperactivity of humoral immunity and neutrophil infiltration were blocked. Meanwhile, the Th1-type immune response and the direct killing function of CD8+ T cells are retained, establishing a bidirectional regulatory model of “anti-inflammation - defense protection”. The reconstruction of this immune homeostasis is highly consistent with the prediction results of network pharmacology. HP targets 40 core genes such as EGFR, GSK3B, and ALOX15, interferes with the NF-κB, IL-17, and TNF signaling axes, and forms a multi-level regulatory network of “gene - protein - pathway”. The research not only clarified the molecular basis of HP in the treatment of constipation, but also provided a theoretical basis for the development of new postbiotic preparations with both intestinal protection and immune regulation functions, suggesting its translational medical potential to achieve organ homeostasis regulation through epigenetic modification and microenvironment remodeling.

## Data Availability

The original contributions presented in the study are included in the article/**Supplementary Material**. Further inquiries can be directed to the corresponding authors.

## Supplementary material

Supplementary Table 1 16s rRNA sequence of *Lactobacillus paracasei.*

Supplementary Table 2 URLs of databases related to network pharmacology analysis.

Supplementary Table 3 significant PANoptosis-related genes in HP treatment of constipation. EGFR, GSK3B, ALOX15, CDK1, AXL, XIAP, MAPT, TNF, PARP1, BIRC3, BIRC2, DRD2, HSP90AA1, HSP90AB1, MAPK14, MTOR, IKBKE, BCL2, CASP3, CASP6, CASP7, CASP1, EZH2, DNMT1, SIRT1, CASP8, STAT3, KIF11, CDK9, TP53, TLR4, NFE2L2, NR4A1, RELA, BRD4, NFKB1, STING1, TRIM24, SIRT3, JUN.

Supplementary Figure 1 Characterization of T-lymphocyte subpopulations and cytokine secretion in the mouse spleen. Frequency of CD8+ & IFN-r+, CD8+ & IL-2+ in the spleens of each group of mice using flow cytometry. NS; * P<0.05; ** P<0.01; *** p<0.001.

Supplementary Figure 2 Postbiotic of hawthorn-probiotic alleviates LPS-induced inflammation and apoptosis in intestinal epithelial cells. (A) The mRNA expression levels of caspase3, caspase9 in NCM460 cells. (B) The mRNA expression levels of TNF-α, TNFR1 in NCM460 cells. (C) The mRNA expression levels of caspase3 in CaCO2 cells. (D) The mRNA expression levels of TNF-α in CaCO2 cells. N:cell treated with culture medium. M: cell treated with 10ug/ml LPS. HP: cell treated with 5% postbiotic of hawthorn-probiotic.NS; * P<0.05; ** P<0.01; *** p<0.001.
