# Supplementary material for: The postbiotic of hawthorn-probiotic ameliorates constipation by multi-pathway inhibition of PANoptosis in intestinal epithelial cells
Source: Front Immunol. 2025 Sep 19;16:1622619. doi: 10.3389/fimmu.2025.1622619 (PMC12492494; doi:10.3389/fimmu.2025.1622619)
Supplement: Supplementary Table 2 — URLs of databases related to network pharmacology analysis. [file Table2.docx]

EGFR, GSK3B, ALOX15, CDK1, AXL, XIAP, MAPT, TNF, PARP1, BIRC3, BIRC2, DRD2, HSP90AA1, HSP90AB1, MAPK14, MTOR, IKBKE, BCL2, CASP3, CASP6, CASP7, CASP1, EZH2, DNMT1, SIRT1, CASP8, STAT3, KIF11, CDK9, TP53, TLR4, NFE2L2, NR4A1, RELA, BRD4, NFKB1, STING1, TRIM24, SIRT3, JUN
