# Supplementary material for: The postbiotic of hawthorn-probiotic ameliorates constipation by multi-pathway inhibition of PANoptosis in intestinal epithelial cells
Source: Front Immunol. 2025 Sep 19;16:1622619. doi: 10.3389/fimmu.2025.1622619 (PMC12492494; doi:10.3389/fimmu.2025.1622619)
Supplement: Supplementary Table 3 — significant PANoptosis-related genes in HP treatment of constipation. EGFR, GSK3B, ALOX15, CDK1, AXL, XIAP, MAPT, TNF, PARP1, BIRC3, BIRC2, DRD2, HSP90AA1, HSP90AB1, MAPK14, MTOR, IKBKE, BCL2, CASP3, CASP6, CASP7, CASP1, EZH2, DNMT1, SIRT1, CASP8, STAT3, KIF11, CDK9, TP53, TLR4, NFE2L2, NR4A1, RELA, BRD4, NFKB1, STING1, TRIM24, SIRT3, JUN. [file Table3.docx]

| **Database Name** | **URL** |
| --- | --- |
| SwissTargetPrediction | https://www.swisstargetprediction.ch/ |
| SuperPred | https:/ /prediction.charite.de/ |
| UniPort | https://www.uniprot.org/ |
| OMIM | https://www.omim.org |
| GeneCards | https://www.genecards.org |
| STRING | http://stringdb.org |
| DAVID | https://davidbioinformatics.nih.gov/ |
| Wei Sheng Xin | https://www.bioinformatics.com.cn/ |
