# Supplementary figures and images for: The postbiotic of hawthorn-probiotic ameliorates constipation by multi-pathway inhibition of PANoptosis in intestinal epithelial cells
Source: Front Immunol. 2025 Sep 19;16:1622619. doi: 10.3389/fimmu.2025.1622619 (PMC12492494; doi:10.3389/fimmu.2025.1622619)

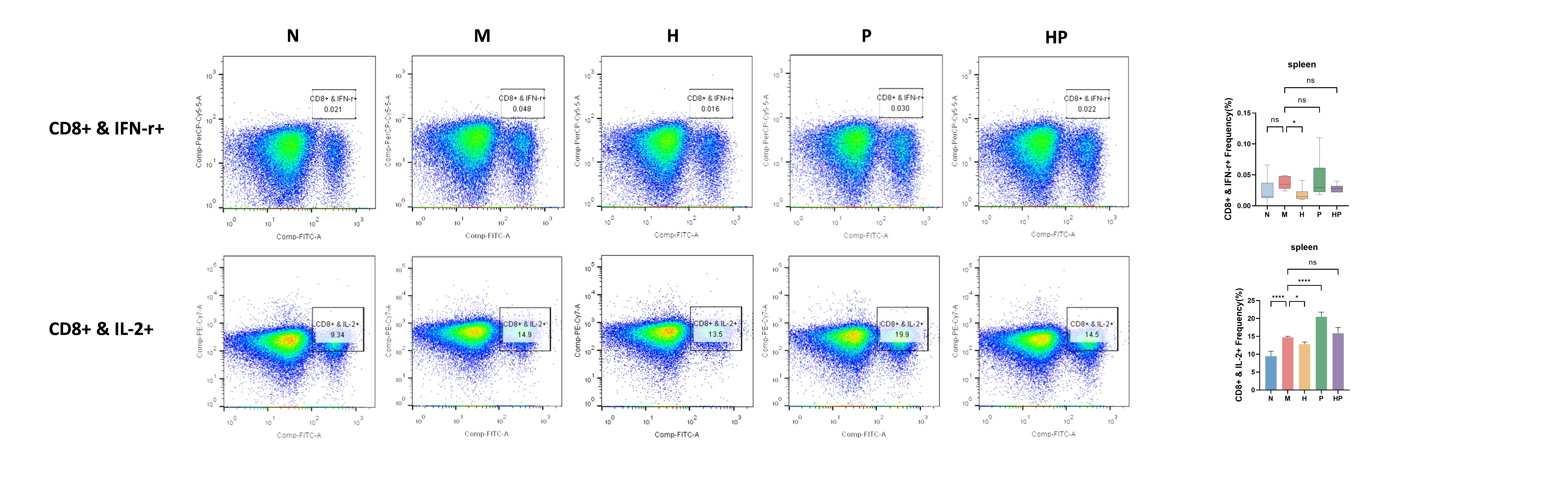

Supplement: Supplementary Figure 1 — Characterization of T-lymphocyte subpopulations and cytokine secretion in the mouse spleen. Frequency of CD8+ & IFN-r+, CD8+ & IL-2+ in the spleens of each group of mice using flow cytometry. NS; * P<0.05; ** P<0.01; *** p<0.001. [file Image1.jpeg]

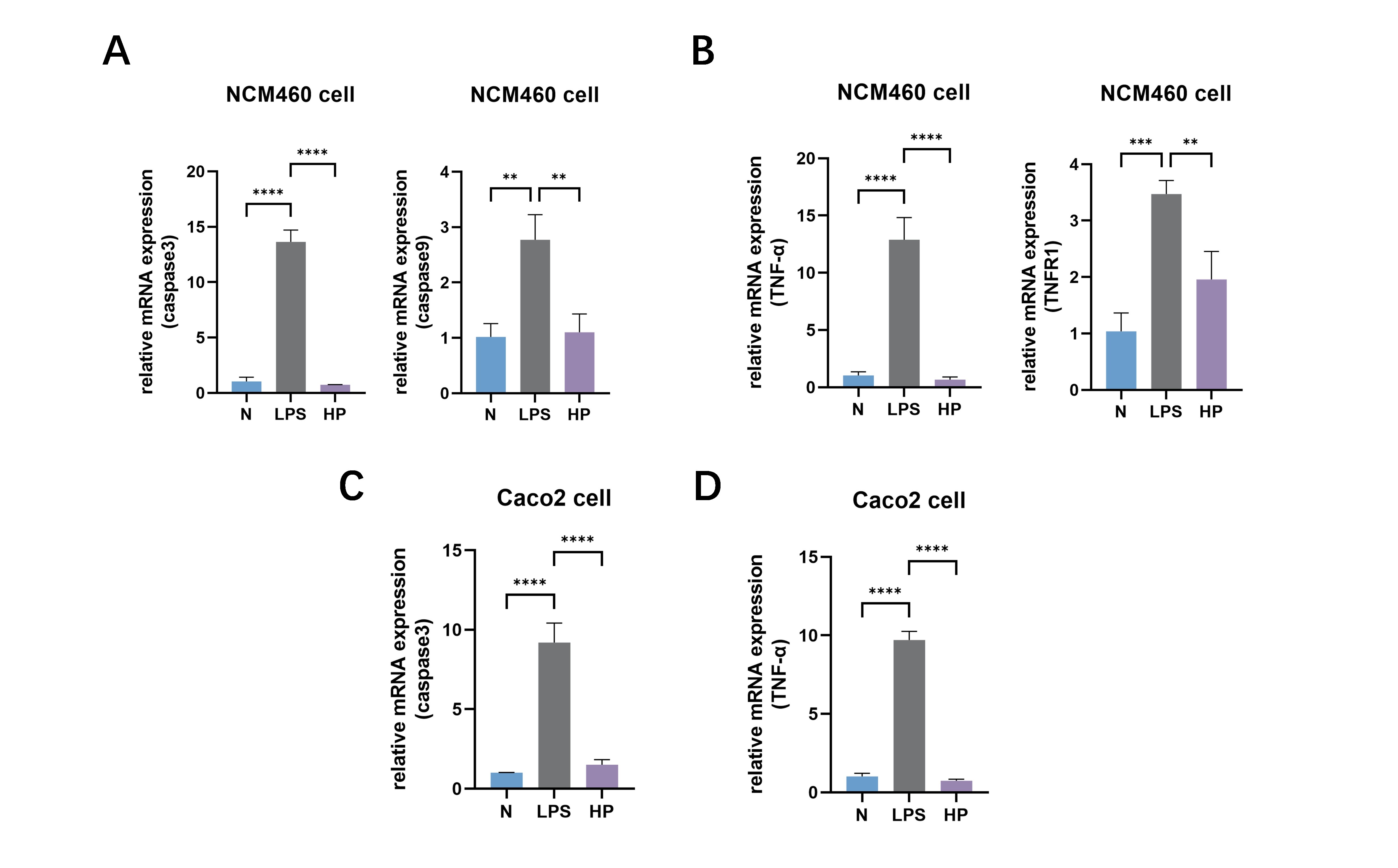

Supplement: Supplementary Figure 2 — Postbiotic of hawthorn-probiotic alleviates LPS-induced inflammation and apoptosis in intestinal epithelial cells. (A) The mRNA expression levels of caspase3, caspase9 in NCM460 cells. (B) The mRNA expression levels of TNF-α, TNFR1 in NCM460 cells. (C) The mRNA expression levels of caspase3 in CaCO2 cells. (D) The mRNA expression levels of TNF-α in CaCO2 cells. N:cell treated with culture medium. M: cell treated with 10ug/ml LPS. HP: cell treated with 5% postbiotic of hawthorn-probiotic.NS; * P<0.05; ** P<0.01; *** p<0.001. [file Image2.jpeg]
